# How Does Experience Modulate Auditory Spatial Processing in Individuals with Blindness?

**DOI:** 10.1007/s10548-013-0339-1

**Published:** 2013-12-10

**Authors:** Qian Tao, Chetwyn C. H. Chan, Yue-jia Luo, Jian-jun Li, Kin-hung Ting, Jun Wang, Tatia M. C. Lee

**Affiliations:** 1 Applied Cognitive Neuroscience Laboratory, Department of Rehabilitation Sciences, The Hong Kong Polytechnic University, Hung Hom, Kowloon, Hong Kong, China; 2 National Key Laboratory of Cognitive Neuroscience and Learning, Beijing Normal University, Beijing, China; 3 China Rehabilitation Research Center, Beijing, China; 4 Laboratory of Neuropsychology, Department of Psychology, The University of Hong Kong, Pokfulam, Hong Kong, China; 5 Institute of Clinical Neuropsychology, The University of Hong Kong, Pokfulam, Hong Kong, China

**Keywords:** Cross-modal plasticity, Sound localization, Superior frontal gyrus, Middle occipital gyrus

## Abstract

Comparing early- and late-onset blindness in individuals offers a unique model for studying the influence of visual experience on neural processing. This study investigated how prior visual experience would modulate auditory spatial processing among blind individuals. BOLD responses of early- and late-onset blind participants were captured while performing a sound localization task. The task required participants to listen to novel “Bat-ears” sounds, analyze the spatial information embedded in the sounds, and specify out of 15 locations where the sound would have been emitted. In addition to sound localization, participants were assessed on visuospatial working memory and general intellectual abilities. The results revealed common increases in BOLD responses in the middle occipital gyrus, superior frontal gyrus, precuneus, and precentral gyrus during sound localization for both groups. Between-group dissociations, however, were found in the right middle occipital gyrus and left superior frontal gyrus. The BOLD responses in the left superior frontal gyrus were significantly correlated with accuracy on sound localization and visuospatial working memory abilities among the late-onset blind participants. In contrast, the accuracy on sound localization only correlated with BOLD responses in the right middle occipital gyrus among the early-onset counterpart. The findings support the notion that early-onset blind individuals rely more on the occipital areas as a result of cross-modal plasticity for auditory spatial processing, while late-onset blind individuals rely more on the prefrontal areas which subserve visuospatial working memory.

## Introduction

Information received by sensory systems needs to be processed and integrated before it can be meaningfully utilized by individuals (Beer et al. 2011). Processing of sensory information can be modulated by an individual’s experience in life. For instance, the lack of visual input among congenitally blind individuals has been revealed to alter their processing of spatial information resulting in under-development of spatial knowledge (Emier 2004; Rieser et al. 1992). The present study explored the mechanisms behind prior visual experience modulating auditory spatial processing. The neural processes associated with sound localization were compared between individuals with early- and late-onset blindness. The findings can shed light on the role of visuospatial function in auditory spatial processing and cross-modal plasticity involving the visual system.

Previous studies investigating functional differences between early- and late-onset blind individuals include pitch change discrimination (Kujala et al. 1997), tactile discrimination (Sadato et al. 2002), Braille reading (Büchel et al. 1998; Cohen et al. 1999), and auditory spatial processing (Collignon et al. 2013; Voss et al. 2008, 2011). A common theme revealed across these studies is the differentiation of involvement of the occipital areas between the two groups. Individuals who were early-onset blind were consistently found to recruit more occipital areas, particularly the primary visual cortex (V1), than their late-onset blind counterparts in non-visual tasks (e.g., Burton et al. 2002a, b). Another between-group difference was the cross-modal connectivity with the occipital cortex. Functional connectivity studies reported that congenitally and early-onset blind individuals appeared to rely on a direct feed-forward cortico-cortical connection, whereas late-onset blind individuals relied on a feed-back cortico-cortical connection for mediating non-visual processing (Collignon et al. 2013; Wittenberg et al. 2004).

Auditory spatial processing is crucial in the everyday lives of individuals with blindness, for example, when navigating within a space and orienting oneself to a person in conversation. Voss et al. (2008) revealed, when compared with the late-blind, the early-blind group performed better in discriminating monaural sounds. Increases in BOLD responses in the middle frontal gyrus and right parietal cortex were found to associate with the discrimination process in both groups. However, the superior discrimination ability of the early-blind group was found to correlate with increased cerebral blood flow in the left dorsal extrastriate cortex, which included the middle occipital gyrus. Collignon et al. (2013) in a recent study investigated the role of experience in shaping functional organization of the occipital cortex during processing of pitch or spatial attributes of sounds. The main difference in auditory spatial processing between congenitally blind and late-onset blind participants was that the former showed increased BOLD responses in the right dorsal stream, which included the middle occipital gyrus and cuneus. Collignon et al. (2013) proposed that the functionality of the dorsal stream among early-blind individuals was for spatial computations of inputs from a non-visual system. More importantly, this cross-modal functional specialization was likely to be developed only early in life.

The notion of prior visual experience modulating auditory spatial processing is interesting in two ways. First, prior visual experience would interfere with ways in which auditory spatial information is processed among individuals with blindness. Among individuals with normal vision, visual experience provides basic pictorial information for spatial processing (Emier 2004; Mark 1993). The inferior parietal lobule has been found to mediate such process (Macaluso and Driver 2005). Auditory spatial processing inevitably would couple with visuospatial working memory, particularly when the information itself or the processes involved become complex (Arnott and Alain 2011; Lehnert and Zimmer 2006, 2008; Martinkauppi et al. 2000). Studies revealed that the parieto-frontal network was associated with auditory spatial processing among late-onset blind individuals, suggesting possible involvement of visuospatial working memory in these processes (Courtney et al. 1996; Ricciardi et al. 2006). The key neural substrates of this network are in the dorsolateral regions of the prefrontal cortex, particularly the middle frontal gyrus and superior frontal gyrus. In contrast, early-onset blind individuals would be less inclined to involve visuospatial function, which is under-developed (Cornoldi and Vecchi 2000; Thinus-Blanc and Gaunet 1997; Vecchi et al. 1995), in auditory spatial processing. Second, without prior visual experience, early-onset blind individuals are likely to rely on non-visual systems for mediating auditory spatial processing. Previous studies have revealed an extensive occipito-parietal network in congenitally and early-onset blind individuals while processing auditory spatial information (e.g., Collignon et al. 2011; Renier et al. 2010; Weeks et al. 2000). Chan et al. (2012) reported a parieto-frontal network mediating auditory spatial processing in a distance judgment task among congenitally blind individuals.

This study aimed to understand how prior visual experience would modulate the auditory spatial processing among blind individuals. The prior visual experience was tested by comparing two groups of blind individuals (early- vs. late-onset blindness) with different levels of visual experience (in years). Different from previous studies, this study used a sound localization paradigm which combines depth into the distances and the sound localization can only be resolved by using subtle spectral cues embedded in the auditory stimuli. The auditory stimuli were simple *da*–*da*–*da* sounds recorded from the electronic “Bat-ears.” “Bat-ears” is a non-invasive, ultrasonic emission device developed for assisting navigation of individuals with blindness. The ultrasound pulses that reflected from obstacles placed in different locations are collected, amplified, demodulated, and put out as audible signals through earphones. In other words, the auditory stimuli were recordings of previous played sounds containing the echo cues created by obstacles at different spatial locations rather than sounds emitted from spatially distinct areas. In this way, the audible sounds contained spatial information with pitch and intensity indicating azimuth and distance (Blumsack and Ross 2007). The experimental task used required the participants to localize each sound stimulus on a 15-location fan-shape space (5 azimuths × 3 distances) which ensures auditory spatial localization process. The findings obtained can shed light on the neural processes associated with auditory spatial localization, as well as help validate usefulness of the “Bat-ears” as a navigation device for people with blindness. With prior visual experience, we hypothesize that the late-onset blind individuals would involve visuospatial process during sound localization which is reflected from increase in BOLD responses in neural substrates mediating such processes. In contrast, sound localization of early-onset blind individuals would rely on cross-modal plasticity involving the occipital cortex. To further address the possibility of involving visuospatial function in auditory spatial processing, tests of visuospatial working memory and general intellectual abilities were administered to the participants, which formed the behavioral correlates of the study.

## Materials and Methods

### Participants

The early-onset blind group was composed of 15 participants (mean age: 28.9 years; from 20 to 38 years) who lost vision at birth (n = 11), or before 1 year of age (n = 3), or at 1 year old (n = 1) (Table 1). The late-onset blind group was composed of 17 participants (mean age: 32.4 years; from 20 to 49 years) who lost vision later in life with a mean onset age of 20.4 years and a mean duration of blindness of 12.0 years. At the time of this study, all participants reported no light perception and normal hearing abilities. Hearing abilities were assessed with a pitch discrimination test (Collignon et al. 2007) that employed stimuli resembling those presented in the sound localization task. Participants had to attain an accuracy rate higher than 60 % to satisfy the inclusion criteria. All participants scored within the normal range on the WAIS-RC (Gong 1982; Wechsler 1955). Before the fMRI acquisition, all participants underwent 30 min of familiarization with the sound-to-azimuth and sound-to-distance relationships using a headphone and joystick. It covered six *da*–*da*–*da* sounds (3 azimuths: −30°, 0°, +30° and 2 distances: 1 and 4 m). The study was approved by the Human Ethics Committee of The Hong Kong Polytechnic University and Beijing Normal University. All participants gave their consent by stamping their fingerprint on the consent form. They were remunerated RMB760 (equivalent to US120) to compensate for travel expenses and time loss.

**Table 1:** Demographic characteristic of the blind participants

Subject number	Education	Gender	Age	Etiology	Onset age of blindness
Early-onset blind group (N = 15)					
1	Vocational education	M	20	Congenital glaucoma	Birth
2	Vocational education	F	30	Retintis pigmentosa	Birth
3	Secondary school	F	29	Retintis pigmentosa	1 year
4	High school	M	38	Optic nerve damage	Birth
5	Vocational education	M	28	Congenital cataract	Birth
6	High school	F	20	Congenital optic atrophy	Birth
7	Vocational education	F	26	Congenital optic atrophy	Birth
8	Vocational education	M	31	Congenital cataract	<1 year
9	Vocational education	M	28	Optic nerve damage	Birth
10	Vocational education	F	30	Retintis pigmentosa	Birth
11	Secondary school	M	26	Congenital optic atrophy	Birth
12	High school	F	30	Retintis pigmentosa	<1 year
13	Secondary school	M	32	Congenital glaucoma	Birth
14	Vocational education	M	36	Optic nerve damage	Birth
15	Vocational education	F	30	Congenital glaucoma	<1 year
Late-onset blind group (N = 17)					
1	College	M	32	Optic nerve damage	30
2	Vocational education	M	35	Glaucoma	26
3	Vocational education	M	38	Ocular fundus disease	5
4	College	M	28	Cataract	22
5	High school	M	38	Retinal detachment	36
6	Vocational education	M	29	Glaucoma	18
7	Secondary school	M	49	Optic nerve damage	39
8	Vocational education	M	28	Optic nerve damage	18
9	High school	M	28	Retintis pigmentosa	13
10	High school	M	43	Retintis pigmentosa	33
11	Secondary school	M	34	Optic nerve damage	15
12	Secondary school	M	27	Cataract	15
13	High school	M	32	Retinal detachment	22
14	Vocational education	F	30	Retinal detachment	9
15	Vocational education	M	20	Retintis pigmentosa	9
16	Secondary school	F	27	Retinal detachment	7
17	Secondary school	M	33	Retinal detachment	30

### Behavioral Test—Matrix Test

The adapted matrix test (Cornoldi et al. 1991) was administered to assess the participants’ visuospatial working memory. There were two haptic subtests: one 2D matrix (3 × 3 squares) comprised of 9 wooden cubes (2 cm per side) and one 3D matrix (2 × 2 × 2 squares) comprised of eight wooden cubes (2 cm per side). Each participant was to mentally maneuver a designated target on the surface of the matrix according to verbal scripts. In each trial, the starting position of the target was presented to the participant by means of a sandpaper pad attached to the surface of a designated square on the matrix. The participant was to tactually recognize and memorize the location of the target. The participant then heard instructions for relocating the target, such as forward–backward and right–left for the 2D matrix, or forward–backward, right–left, and up–down for the 3D matrix. The relocation instructions were delivered to the participant using a tape recorder. To demonstrate performance with a moderate level, we manipulated 2–3 targets together with 2–4 steps of relocation instructions in each trial. After playback of the instructions, the participant was given the blank matrix without targets, which indicated the terminal location of the imagery target. There were 12 trials in each of the 2D and 3D matrices. The performance scores were the percentages of the accurate terminal locations indicated by the participant.

### Behavioral Test—Intelligence Test

Most studies indicated that the verbal intelligence performance of visually impaired individuals was comparable to their sighted counterparts on the Wechsler Adult Intelligence Scale (WAIS) (Vander Kolk 1977, 1982). To evaluate intelligence of the blind participants, the WAIS-RC) (Gong 1982; Wechsler 1955) was administered to each participant by an examiner. The WAIS-RC has been validated using factor analysis in a large sample of Chinese urban (N = 2029) and rural (N = 992) residents (Dai et al. 1990). Since then, the WAIS-RC has been widely used in educational and clinic settings in China. The WAIS-RC verbal test (Gong 1982; Wechsler 1955) included six subtests: information, vocabulary, comprehension, similarities, digit span, and arithmetic. All tests were conducted verbally by the examiner following the standardized procedures.

### fMRI Tasks

**Fig. 1:**
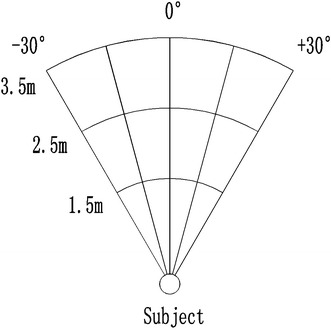
A *fan-shape space* indicating the locations from which the “Bat-ears” sounds were emitted (in a sound proof chamber). There were 15 locations, including five azimuths (−30° [left side], −15°, 0°, +15°, +30° [right side]) and three distances (1.5, 2.5, 3.5 m)

The auditory stimuli were similar to those fabricated in Chan et al. (2012), which were generated from the “Bat-ears” device. Ultrasonic pulses were emitted from a generator located in the center of the “Bat-ears.” The pulses, when meeting a designated obstacle, were reflected as echoes, which were captured by the binaural receivers mounted on the two sides of the “Bat-ears.” These ultrasound echoes were converted to *da*–*da*–*da* sounds (peak frequencies 3,200–4,700 Hz) and recorded by a KEMAR Manikin, on which the “Bat-ears” were placed (Burkhard and Sachs 1975). The entire procedure was conducted in an acoustic laboratory. The obstacle was made of an erected piece of cardboard (30 × 30 cm) located at specific designations in the sound-proof chamber. The “Bat-ears” and the center of the obstacle were placed at a height of 1.5 m. The designations were organized in a fan-shape space that was organized into five azimuths (−30° [left side], −15°, 0°, +15°, +30° [right side]), and three distances (1.5, 2.5, and 3.5 m from the “Bat-ears” and Manikin) (Fig. 1). There were a total of 15 locations from which the echoes were reflected and recorded as the *da*–*da*–*da* stimuli which were of higher resolution than those recorded from three locations as in Chan et al. (2012) study.

**Fig. 2:**
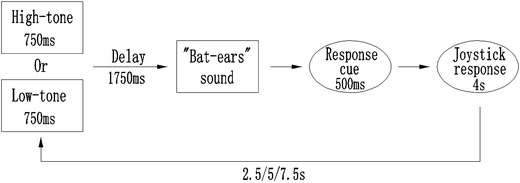
The sound localization paradigm with auditory spatial processing occurred during the presentation of the 3-s “Bat-ears” sound. For each trial, an auditory cue was presented for 750 ms to indicate the task type: the localization task trial was indicated by a high-tone (peaked at 2,000 Hz) and the differentiation control trial was indicated by a low-tone (peaked at 500 Hz). After 1,750 ms delay, the 3-s auditory stimulus was presented and followed by a 500-ms cue to indicate the preparation for response. Finally, the left time was for the joystick response

**Fig. 3:**
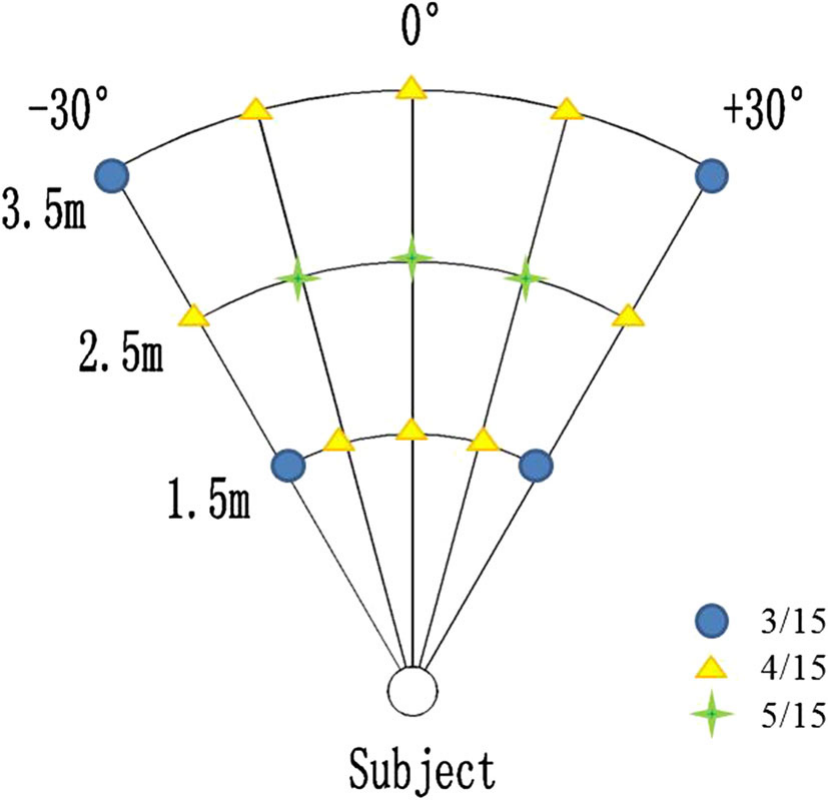
Definition for correct responses. Neighboring positions having the same azimuth or distance as the exact correct positions but with one-step difference in distance or azimuth are also regarded as correct responses. Therefore, the locations indicated as *blue circles* (e.g., −30°/1.5 m) each has two neighboring positions as correct responses indicated as *yellow triangles* (e.g., −30°/2.5 m or −15°/1.5 m); the locations indicated as *green stars* (e.g., +15°/2.5 m) each has four neighboring positions as correct responses. The chance level of accuracy for the *blue, yellow* and *green* locations are 20, 26.67, and 33.33 % respectively. The lenient criteria used would lower difficulty level of the sound localization task which increases power of the analyses (Color figure online)

The sound localization task required the participant to listen to the “Bat-ears” stimulus (peak frequencies 3,200–4,700 Hz, 70 dB) and identify the location on the 5 azimuths × 3 distances space from which the sound would have been emitted. The task process required the participant to listen to the stimulus and extract the spatial information embedded in the sound (such as intensity and frequency). Based on the information, the participant was to estimate the location of the sound source and indicate it using a joystick with the right hand. The participant made a response by maneuvering the joystick to one location, which indicated both azimuth and distance of the sound source. The calibration of the joystick was: left/straight/right indicated −15°/0°/+15°, and outer left/right indicated −30°/+30°; backward/horizontal/forward indicated 1.5/2.5/3.5 m. So a joystick position of forward-outer-left would mean a sound location of −30° at 3.5 m. The control task was a pitch discrimination task that required the participant to differentiate whether the “Bat-ears” sound had been inserted with a 15-ms sound clip of a different pitch (6,000–8,000 Hz, 70 dB). The task process was to listen to the sound and extract its specific frequency information. The participant judged whether the sound had or did not have an inserted pitch. A “Yes” or “No” response was made by pressing or by not pressing on the joystick, respectively. The discrimination control task would produce baseline BOLD responses associated with non-spatial auditory processing of the “Bat-ears” stimuli. The auditory stimuli were the same in both the sound localization and pitch differentiation tasks, which could control for possible confounding factors associated with the physical attributes of the stimuli. For each trial, an auditory cue was presented for 750 ms to indicate the task type: localization (2,000 Hz, 70 dB) or differentiation (500 Hz, 70 dB). There was a 1,750 ms delay, during which the participant was to orientate himself to the task and recall its process and requirement (Fig. 2). The *da*–*da*–*da* stimulus was presented for 3,000 ms, which was followed by a 500 ms auditory cue (2,000 Hz, 70 dB) for the participant to prepare to make the response with the joystick. The time available for response was 4,000 ms. The inter-trial interval (ITI) was set at 12,500/15,000/17,500 ms, with a uniform distribution of jitters (2,500, 5,000, or 7,500 ms). Response time was not used as a behavioral measure because localization responses at a farther distance (e.g., 3.5 m) and at the outer left/right side (e.g., ±30°) took a longer time to register on the joystick than those at a closer distance (e.g., 1.5 m) and at the center (e.g., 0°). Performance for the localization task was measured in terms of the accuracy of the location of the sound source estimated by the participant in the localization task trials. The participants in general found that localizing the sounds required some effort particularly when the task was carried out in the scanner. Lenient criteria, i.e. localization of the exact correct or neighboring positions, were applied to defining correct trials. For instance, responses at two neighboring locations were regarded as “correct” for localizing a stimulus emitted from the outer-left farthest-distance location (−30°, 3.5 m). They were the outer-left medium-distance (−30°, 2.5 m) or left farthest-distance (−15°, 3.5 m). Therefore, the chance level for different positions was varied from 20 to 33.33 % (Fig. 3).

### fMRI Data Acquisition

The auditory stimuli were bilaterally presented via an MRI compatible headphone system, and the sound-pressure level of the stimuli was adjusted from 70 to 80–90 dB to compensate for the noisy environment inside the scanner. Each participant was scanned in four fMRI runs using an event-related design. In each run, the number of the sound localization/differentiation trials was unbalanced, with 17–20 localization task trials and 8–11 differentiation control trials. The order of the runs was counterbalanced among the participants. These gave a total of 75 localization task trials and 37 differentiation control trials. The fMRI series were captured by a 3-T Siemens machine with a 12-channel head coil. Functional T2^*^ images were obtained with a gradient echo-planar sequence (repetition time [TR] = 2,500 ms; echo time [TE] = 30 ms; flip angle [FA] = 90°; voxel size = 3.1 × 3.1 × 3.2 mm^3^). Structural T1 images (TR = 2,530 ms; TE = 3.39 ms; voxel size = 1.3 × 1.0 × 1.3 mm^3^) were also acquired.

### fMRI Image Analysis

Analyses were carried out using SPM8 (Welcome Department of Imaging Neuroscience, London, UK), implemented in MATLAB (Mathworks). Preprocessing included slice timing for correcting differences in the timing of acquisition between slices, realignment of functional time series for correcting head motion, coregistration of functional and anatomical data, segmentation for extracting grey matter, spatial normalization to the Montreal Neurological Institute (MNI) space, and spatial smoothing (Gaussian kernel, 6 mm FWHM).

The preprocessed data were fitted to a general linear model (GLM) in SPM8 (Friston et al. 1994) using two event-related regressors. The two regressors modeled the BOLD signals corresponding to the correct responses made in the localization task trials and differentiation control trials, which were constructed by convolving the onset times of the “Bat-ears” sound with the canonical hemodynamic response function. The motion parameters detected by the Artifact Detection Tools (ART, developed by the Gabrieli Lab, Massachusetts Institute of Technology, available at: http://web.mit.edu/swg/software.htm) were included in the GLM for further regression of the motion-dependent confound (Mazaika et al. 2005). Slow changes in the data were removed by applying a high-pass filter with a cut-off of 128 s, and a first-order autoregressive process was used to correct for autocorrelation of residual signals in the GLM.

### Whole Brain and Region-of-Interest Analyses

Whole-brain analyses were first conducted separately for the early- and late-onset blind groups. The contrast of interest involved comparing correct responses of the localization task trials and of the differentiation control trials, and the linear contrast tested the main effect of interest (localization > discrimination). One-sample *t*-tests with random effects (Holmes and Friston 1998) were performed. The statistical threshold for the *t*-images was *P* < 0.05, corrected for family-wise error (FWE corrected) at the voxel level. Two-sample *t*-tests and cluster-level inference (Friston et al. 1996) were then performed to identify group differences between the early- and late-onset blind groups. The thresholds for the *t*-images were *P* < 0.001 (uncorrected) at the voxel level and *P* < 0.05 (FWE corrected) at the cluster level. All of the significant BOLD responses were overlaid on the structural template in MNI space, as provided in SPM8. The automated anatomical labeling (AAL) method was used to label the peak coordinates of the activation clusters (Tzourio-Mazoyer et al. 2002).

To answer the question of how visual experience would modulate the auditory spatial processing, an exploratory region of interest (ROI) analysis was performed on the basis of the current results. The ROIs were defined in two ways: (1) conjunction analysis (Nichols et al. 2005)—common BOLD responses between the two groups of participants with threshold of *t*-image set at *P* < 0.05 (FWE corrected) at the voxel level; and (2) two-sample *t* test analysis—between-group BOLD responses with thresholds of *t*-image set at *P* < 0.001 (uncorrected) at the voxel level and *P* < 0.05 (FWE corrected) at the cluster level. All functional ROIs were created with a 9-mm radius spherical mask centered at the local peaks of the activation clusters. For the ROIs that were identified by the conjunction analysis, two-sample *t*-tests were conducted to identify possible difference in BOLD responses between the two participant groups. Stepwise linear regression was conducted on all ROIs to identify the extent to which the mean contrast values of ROIs predicted performance on the localization task for each of the two participant groups. Pearson’s product-moment correlations were obtained between mean contrast values of all ROIs and onset age of late-onset blind participants and scores on the adapted matrix test for the late-onset blind participants, respectively.

## Results

### Behavioral—fMRI Tasks

In the early-onset blind group, the mean accuracy rate for the sound localization task was 46.8 % (*SD* = 3.8 %). In the late-onset blind group, the mean accuracy rate was 43.6 % (*SD* = 4.4 %). Participants with early-onset blindness had a significantly higher accuracy rate than their late-onset blindness counterparts (*t*(30) = 2.21, *P* = 0.035). It was noteworthy that the participants in both groups performed above the chance level of 33.33 %. The performance on the pitch discrimination task was found comparable between the early- and late-onset blind groups (*t*(30) = 0.18, *P* = 0.86).

### Behavioral—Matrix Test

The number of correct trials on the 2D and 3D subtests of the matrix test were counted. The results for one early-onset and two late-onset blind participants were excluded from analysis as they were found unable to perform the task. The final sample size for the matrix test was 14 for the early-onset and 15 for the late-onset blind group. In the early-onset blind group, the mean accuracy rate for the 2D subtest was 43.3 % (*SD* = 25.2 %); for the 3D subtest, it was 41.9 % (*SD* = 26.4 %). In the late-onset blind group, the mean accuracy rate for the 2D subtest was 54.7 % (*SD* = 13.5 %), and for the 3D subtest, it was 45.8 % (*SD* = 13.5 %). The results did not reveal significant difference in the accuracy rates of the 2D subtest (*t*(19.58) = −1.49, *P* = 0.15) and 3D subtest (*t*(19.06) = −0.49, *P* = 0.63) between the early- and late-onset blind groups.

### Behavioral—Intelligence Test

Similarly, the results for one early-onset and two late-onset blind participants were regarded as invalid due to non-compliance observed during the testing. The final sample size for the intelligence test was 14 for the early-onset and 15 for the late-onset blind group. Raw scores on each subtest were calculated and converted to the standard scores. Scores on the six subtests were summed and converted to a Verbal IQ score. All participants scored within the normal range (>70) on the Wechsler Adult Intelligence Scale-Revised for China (WAIS-RC) (Gong 1982; Wechsler 1955). The Verbal IQ scores for the early-onset blind group ranged from 74 to 131, with a mean of 102.9 (*SD* = 16.7), and the Verbal IQ scores for the late-onset blind group ranged from 96 to 122, with a mean of 107.5 (*SD* = 7.1). The results did not reveal significant difference in the Verbal IQ performance between the early- and late-onset blind groups (*t*(17.34) = −0.95, *P* = 0.35).

### BOLD Responses Associated with Auditory Spatial Processing

**Fig. 4:**
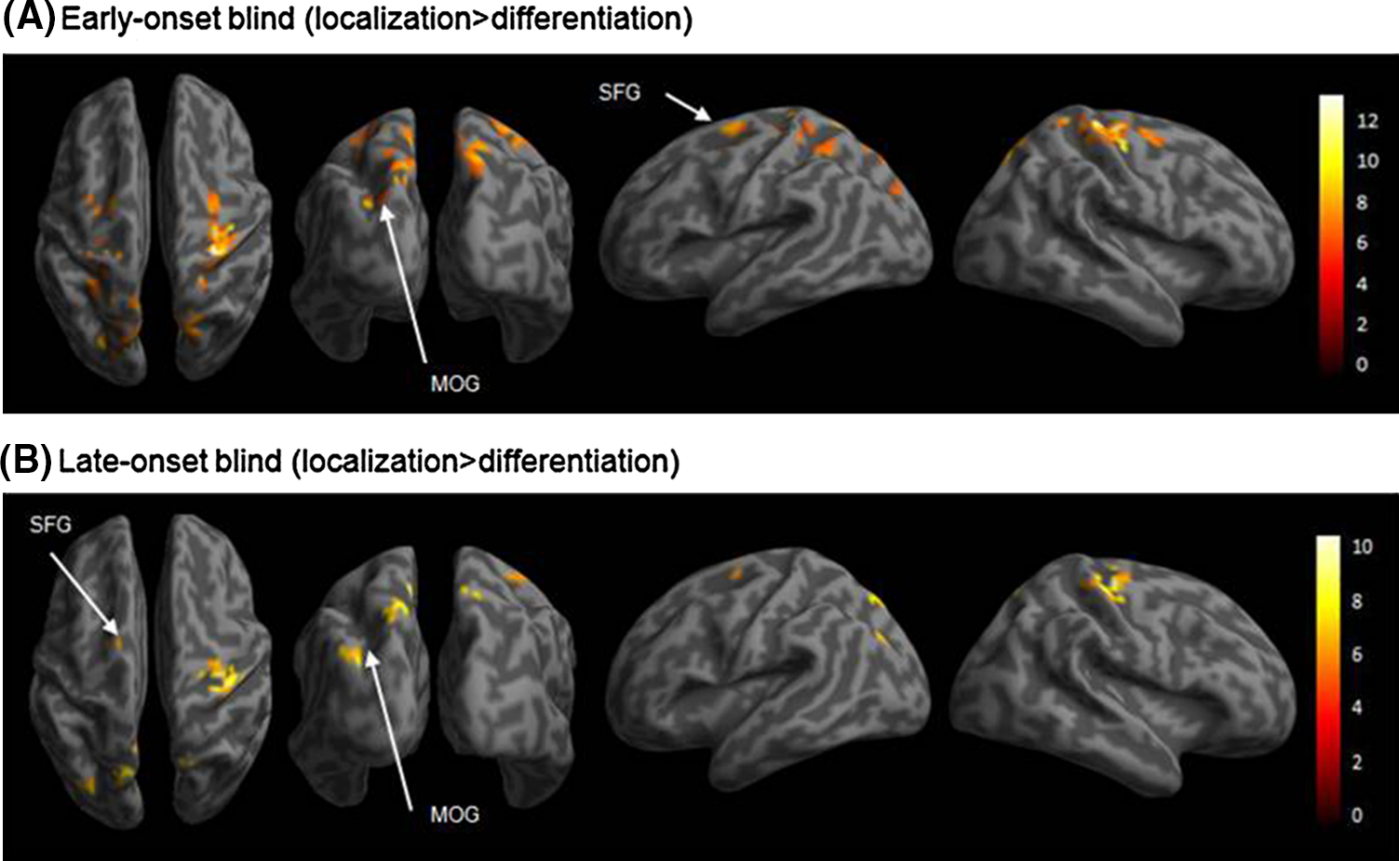
Significant increases in BOLD responses in the contrasts of (localization > differentiation) for the two blind groups. The threshold was *P* < 0.05 (FWE corrected) at the voxel level. **a** The early-onset blind group. Significant increases in BOLD responses were revealed in the left middle occipital gyrus, left precuneus, bilateral superior parietal gyrus, and left superior frontal gyrus. **b** The late-onset blind group. Significant increases in BOLD responses were revealed in the left middle occipital gyrus, left precuneus, and left superior frontal gyrus

**Fig. 5:**
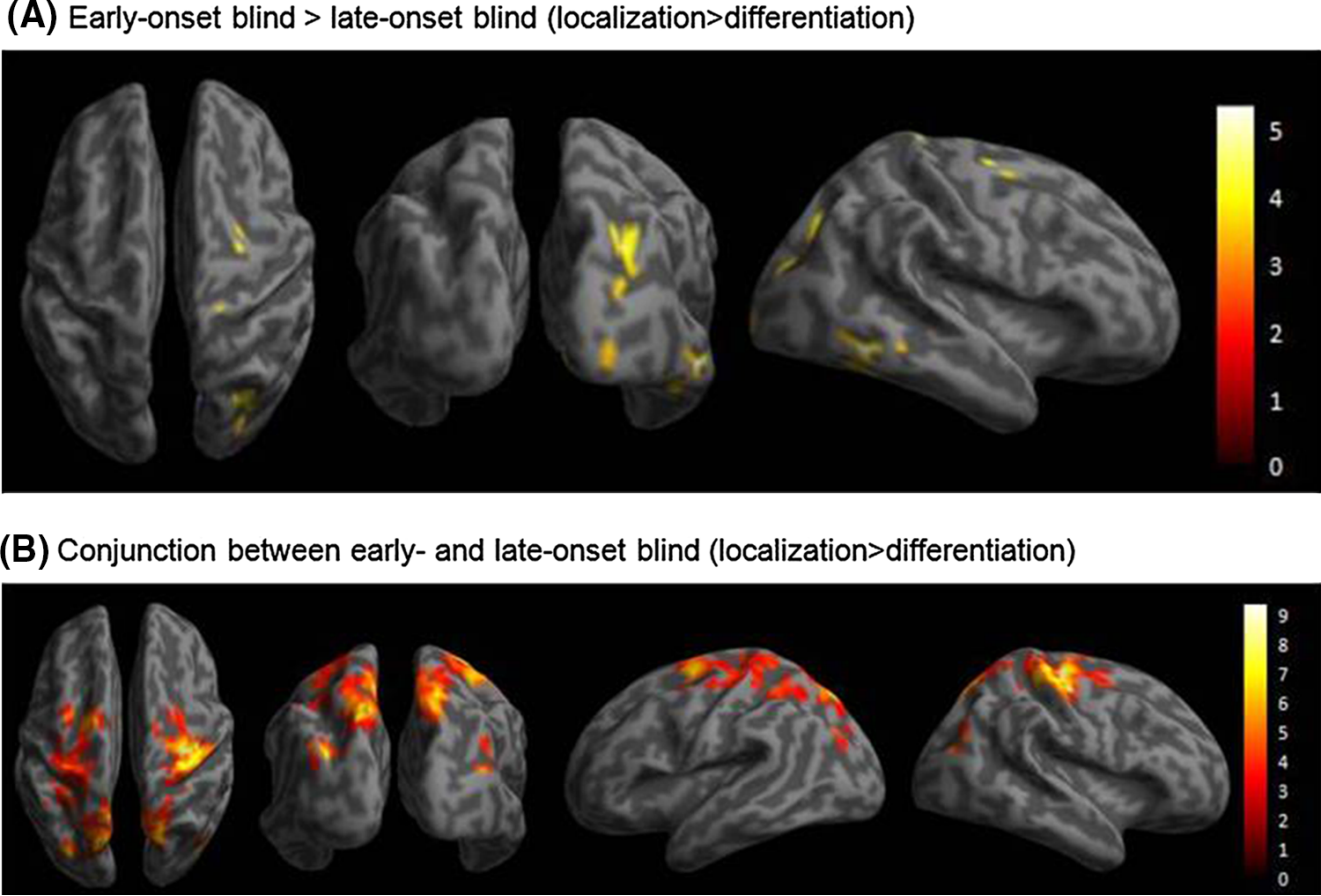
Different and common BOLD responses in the early- and late-onset blind groups. The thresholds are *P* < 0.001 (uncorrected) at the voxel level, and *P* < 0.05 (FWE corrected) at the cluster level. **a** Significant differences in BOLD responses between the two blind groups in the condition: early-onset blind × (localization > differentiation) > late-onset blind × (localization > differentiation). The revealed neural substrates include the right middle occipital gyrus, right lingual gyrus, and right inferior temporal gyrus. **b** Common BOLD responses between the early- and late-onset blind groups in the contrast of (localization > differentiation). The neural substrates include the left middle occipital gyrus, left precuneus, right superior parietal gyrus, and left superior frontal gyrus

Group analyses on the BOLD responses of the linear contrast (localization > discrimination) for the early-onset blind participants (*n* = 15) revealed maxima in the left middle occipital gyrus (MOG), the left precuneus, the bilateral superior parietal gyrus (SPG), the left superior frontal gyrus (SFG), the right supplementary motor area (SMA), the right precentral gyrus, and the right postcentral gyrus (Table 2 [under early-onset blind] and Fig. 4a). A comparable pattern of results was revealed for the late-onset blind participants (*n* = 17). Increases of BOLD responses of the linear contrast (localization > discrimination) were identified in the left MOG, the left precuneus, the left SFG, and the right precentral gyrus (Table 2 [under late-onset blind] and Fig. 4b). Group comparisons revealed that the early-onset blind group had significantly greater BOLD responses than the late-onset blind group in the right inferior temporal gyrus (ITG) and in the occipital cortex, which included the right lingual gyrus (LG), the right MOG, the right superior occipital gyrus (SOG), and the right fusiform gyrus (Table 3; Fig. 5a). Conjunction analysis revealed common BOLD responses across the early- and late-onset blind groups, including the left MOG, the left precuneus, the right SPG, the left SFG, and the right precentral gyrus (Fig. 5b). As the male-to-female ratios were differed in the early- and late-onset blind groups, the gender of participants was tested for its effect on the BOLD responses. Two-sample *t*-tests and conjunction analysis between the two blind groups were repeated with gender as a covariate. The results of the two runs of analyses were comparable except the coordinates of SPG in the conjunction analysis were modified from (18, −69, 52) to (15, −69, 49) (Table 3). It appears that gender would not be a significant factor confounding the results.

**Table 2:** Coordinates, cluster size, and *t*-values of significant BOLD responses for the contrast of (localization > differentiation) in the early- and late-onset blind groups

*x,y,z* (mm)	L/R	Label	Cluster size	*T*	*Z*
Localization > discrimination sounds (early-onset blind)					
−31, −78, 24	L	Middle occipital gyrus	13	9.47	5.22
−10, −75,49	L	Precuneus	14	9.28	5.17
−7, −66, 59	L	Precuneus	6	9.03	5.11
−16, −75, 40	L	Superior parietal gyrus	6	8.64	5.01
−31, −47, 56	L	Superior parietal gyrus	6	8.49	4.97
15, −72, 49	R	Superior parietal gyrus	24	12.59	5.85
−22, −4, 59	L	Superior frontal gyrus	30	10.88	5.53
31, −25, 62	R	Precentral gyrus	108	13.76	6.04
27, −10, 56	R	Precentral gyrus	8	12.03	5.75
34, −44, 62	R	Postcentral gyrus	16	9.65	5.26
6, 3, 52	R	Supplementary motor area	5	10.47	5.44
Localization > discrimination sounds (late-onset blind)					
−31, −81, 33	L	Middle occipital gyrus	16	9.30	5.38
−7, −66, 56	L	Precuneus	62	12.27	6.05
−19, −4, 59	L	Superior frontal gyrus	5	8.26	5.08
34, −25, 59	R	Precentral gyrus	69	11.64	5.92

Coordinates refer to standardized Montreal Neurological Institute (MNI) space. The threshold was *P* < 0.05 (FWE corrected) at the voxel level. (*N* = 15 for the early-onset blind group and* N* = 17 for the late-onset blind group)

**Table 3:** Coordinates, cluster size, and *t*-values of significant BOLD responses for the inter-group contrast between the early- and late-onset blind participants in the contrast of (localization > differentiation)

*x,y,z* (mm)	L/R	Label		Cluster size	*T*	*Z*
Localization > discrimination sounds (early → late-onset blind)						
49, −50, −12	R	Inferior temporal gyrus		68	5.88	4.76
21, −87, −2	R	Lingual gyrus		119	5.42	4.49
34, −66, 27	R	Middle occipital gyrus		82	5.19	4.35
Localization > discrimination sounds (late → early-onset blind)						
NA						

Coordinates refer to standardized Montreal Neurological Institute (MNI) space. The thresholds are *P* < 0.001 at the voxel level and *P* < 0.05 (FWE corrected) at the cluster level. (*N* = 15 for the early-onset blind group and* N* = 17 for the late-onset blind group)

### ROIs: Auditory Spatial Processing

**Fig. 6:**
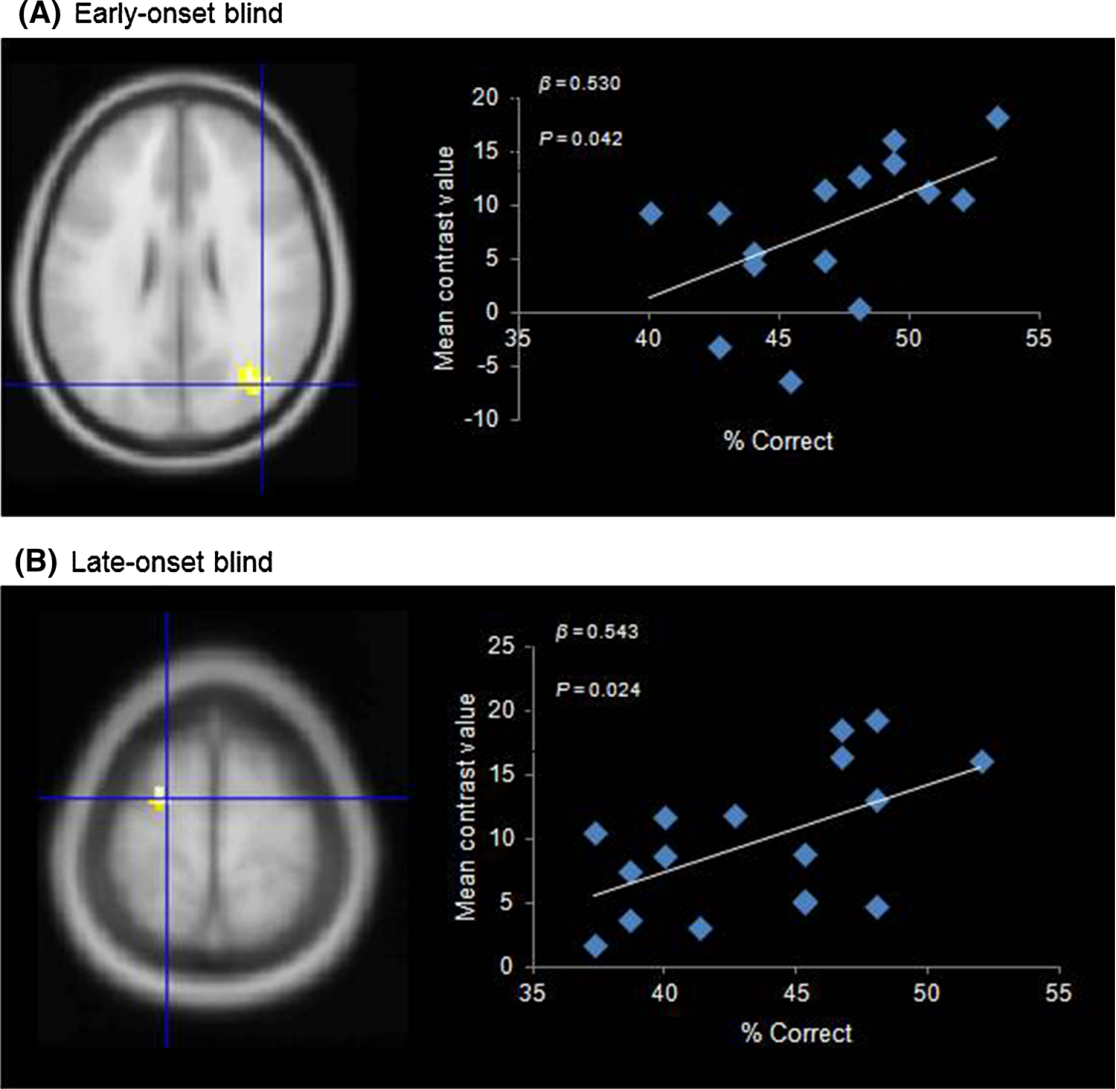
ROI analyses on auditory spatial processing among the early- and late-onset blind groups. Only ROIs that significantly predict the localization performance are presented. The mean contrast values in **a** the right middle occipital gyrus were entered in the regression model for the early-onset blind group, *β* = 0.530, *P* = 0.042; **b** the left superior frontal gyrus were entered in the regression model for the late-onset blind group, *β* = 0.543, *P* = 0.024

Four ROIs were identified from the conjunction analysis: the left MOG, left precuneus, right SPG, and left SFG (Table 4). Three ROIs were identified from the two-sample *t*-test analysis: the right ITG, right MOG, and right LG. Between-group comparisons on the conjunction ROIs (ROIs 1–4) revealed that the early-onset blind group (mean = 20.20) had significantly higher mean contrast values than the late-onset blind group (mean = 13.89) in the right SPG (*t*(30) = 2.47, *P* = 0.02). Regression analyses revealed that only the changes in the mean contrast values in the left SFG significantly predicted performance on the sound localization task (*β* = 0.543, *P* < 0.05) in the late-onset blind participants (Fig. 6). In contrast, only the changes in the mean contrast values in the right MOG significantly predicted localization task performance in the early-onset blind participants (*β* = 0.530, *P* < 0.05). The onsets of blindness of the late-onset blind participants was negatively correlated with the mean contrast values in the left precuneus (r = −0.493, *P* = 0.044) and had a trend for negative correlation with the mean contrast values in the right MOG (r = −0.424, *P* = 0.090). A trend for correlation was found between the duration of blindness and the mean contrast values in the left MOG (r = 0.473, *P* = 0.055). As for the correlations with the visuospatial working memory, we found significant correlations between the mean contrast values in the left SFG and performance on the 2D (r = 0.585, *P* = 0.022) and 3D matrix tests (r = 0.562, *P* = 0.029) among the late-onset blind participants. These were not observed in the early-onset blind participants.

**Table 4:** Coordinates for the ROIs. Some of the ROIs (ROIs1–4) were defined by conjunction analyses, and the threshold is *P* < 0.05 (FWE corrected) at the voxel level

ROI	Label	*x,y,z* (mm)	*T*	*Z*
1	Left middle occipital gyrus	−31, −81, 30	6.95	5.32
2	Left precuneus	−7, −66, 59	8.74	6.12
3	Right superior parietal gyrus	18, −69, 52	7.38	5.53
4	Left superior frontal gyrus	−22, −7, 59	8.03	5.82
5	Right middle occipital gyrus	34, −66, 27	5.19	4.35
6	Right lingual gyrus	21, −87, −2	5.42	4.49
7	Right inferior temporal gyrus	49, −50, −12	5.88	4.76

Coordinates refer to standardized Montreal Neurological Institute (MNI) space. The others (ROIs 5–7) were identified by group comparison analyses, and the thresholds are *P* < 0.001 at the voxel level and *P* < 0.05 (FWE corrected) at the cluster level. (*N* = 15 for the early-onset blind group and* N* = 17 for the late-onset blind group)

## Discussion

The results revealed involvement of the occipital, parietal, and frontal regions during the auditory spatial processing among the late-onset blind participants. Between-group analyses indicated dissociations of neural substrates between the early- and late-onset blind cohorts, which were likely to be attributed to the visual experience gained only by the late-onset blind group. The most significant neural substrates were the right MOG (whole-brain analysis) and the right SPG (ROI analysis). Furthermore, ROI results indicated between-group dissociations in the left SFG and the right MOG. BOLD responses in the left SFG were revealed to associate with performance on the sound localization task among the late-onset blind participants. The role of the SFG in these participants might be attributed to their visuospatial working memory ability, which was reported unique to visual experience. The BOLD responses in the right MOG, in contrast, were revealed to largely mediate auditory spatial processing among the early-onset blind participants who were relatively deprived of visual experience. This was largely in agreement with previous findings on the involvement of the MOG in the spatial analysis of sounds in early-onset blind individuals (Collignon et al. 2011; Renier et al. 2010). Our findings suggested that prior visual experience enhances the involvement of visuospatial processing mediated by the SFG in the late-onset blind individuals. Without prior visual experience, the early-onset blind individuals were found to rely on spatial processing mediated by the MOG for sound localization.

In this study, ROI analyses revealed higher contrast values in the right SPG in the early- than late-onset blind group. This is further supported by the whole brain analyses showing that sound localization was associated with greater BOLD responses in the bilateral SPG, observed only in the early-onset blind group. Neuroimaging studies on blind individuals revealed involvement of the SPG and SPL during auditory spatial processing (Arno et al. 2001; Chan et al. 2012; Gougoux et al. 2005; Sadato et al. 2002). Our results are in accordance with Voss et al. (2008), which showed more SPG recruitment in early-onset blind individuals in discrimination of sound sources. The inconsistent findings of the SPL versus SPG might be attributed to the use of different definitions for labeling neural substrates across studies.

### Functional Specialization of MOG in Auditory Spatial Processing

A group comparison of whole brain analyses revealed more occipital recruitment, particularly from the right MOG during sound localization in the early-onset group (Fig. 2). Stronger occipital responses have been found during cross-modal processing in sound source discrimination (Voss et al. 2008), auditory motion perception (Bedny et al. 2010), Braille reading (Burton et al. 2002a), and language perception (Bedny et al. 2012). Collignon et al. (2013) found more occipital recruitment for auditory processing of pitch and location in the congenitally blind group.

Spatial processing in general is mediated by the dorsal visual pathway (Haxby et al. 1991; Mishkin et al. 1983; Ungerleider and Mishkin 1982). Among various neural structures in the pathway, the MOG has consistently been found to be involved in processing spatial information of different modalities among early-onset blind individuals (Collignon et al. 2009; Dormal and Collignon 2011). For instance, Renier et al. (2010) reported greater BOLD responses in the right MOG when processing spatial information than non-spatial information. Collignon et al. (2011) also demonstrated that, among congenitally blind individuals, preferential BOLD response was observed in the right MOG while processing auditory spatial information over pitch of sounds. The MOG was found to mediate sound localization among individuals with early-onset blindness (Gougoux et al. 2005; Renier et al. 2010). In this study, the changes in mean contrast values in the right MOG of participants in the early-onset group were the only significant predictor of their performance on the sound localization task. The results were consistent with those revealed in previous studies, which supports the notion that the MOG mediates auditory spatial processing among those who had been deprived of visual experience in early life.

It is noteworthy that the late-onset blind participants in this study showed greater BOLD responses in the left MOG during auditory spatial processing. The BOLD responses in the MOG, however, were not found to significantly relate to the behavioral performance. The mean contrast values in the left MOG showed marginal positive correlation with the participants’ duration of blindness. Our results were consistent with Voss et al. (2008, 2011), who reported significant bilateral BOLD responses in the MOG among a group of late-blind individuals. Similarly, the BOLD responses in the MOG appeared to produce no behavioral advantage. Voss et al. (2008) also revealed a significant negative correlation between the BOLD responses in the right MOG and onset age of blindness. The findings seemed to suggest that prior visual experience before blindness influence the structure of and the functions associated with the MOG. The BOLD responses in the MOG appeared to be de-facilitated by the amount of prior visual experience gained by the late-onset blind individuals. Ironically, prior visual experience did not seem to help in preserving the spatial function mediated by the MOG after impairment of the visual system. Our findings lend support to the proposal of a critical period in functional preservation of the dorsal occipital regions for mediating spatial processing among blind individuals (Dormal and Collignon 2011). The critical period would correspond to that of early-onset blind participants of this study, which was within the first year of age.

### Experience-Dependent SFG in Auditory Spatial Processing

Behavioral results showed that the late-onset blind participants performed above the chance level on the sound localization test but performed significantly lower than the early-onset blind participants. This indicated that participants in both groups managed to extract the spatial information embedded in the novel “Bat-ears” sounds for making correct responses. The left SFG was the only neural substrate among the other six ROIs tested that showed significant correlation with the performance on the sound localization task among the late-onset blind participants, but this was not the case with the early-onset blind participants. Therefore, this suggested that the left SFG played a key role in mediating the auditory spatial processing in late-onset blind individuals.

Among the behavioral parameters used in this study, the change in the mean contrast values in the left SFG were found to significantly relate to late-onset blind participants’ scores on the matrix subtests. Such a relationship was not observed in the early-onset blind participants. The matrix test is a measure of visuospatial working memory requiring encoding and retrieval of a series of verbal instructions describing spatial locations. Similarly, encoding of the sound stimuli and retrieving their spatial correlates are one of the critical steps in the sound localization task. Brain imaging studies on visuospatial working memory of late-onset blind individuals cannot be found. Studies on individuals with normal vision revealed that the prefrontal cortex plays a key role in mediating visuospatial working memory (Goldman-Rakic 1994, 1995). Other studies using non-visual tasks revealed recruitment of the dorsal “visual” stream in the dorsolateral prefrontal cortex (Courtney et al. 1996; Nelson et al. 2000). Courtney et al. (1998) further identified the superior frontal sulcus as the main neural substrate mediating spatial working memory. Specifically, the BOLD responses in the medial frontal gyrus, superior frontal sulcus (SFS) and SFG, and intraparietal sulcus were found to be dependent on the memory load required by the tasks. With prior visual experience, the late-onset blind participants in this study would tend to involve visuospatial working memory for processing the spatial information embedded in the “Bat-ears” sounds, such as their intensity and frequency. The decision on the location of the reflected sounds which would induce a memory load is likely to be mediated by the left SFG.

## Limitations

This study has a few limitations. First, the participants were recruited by means of convenience sampling. The results can only be generalized to those who share similar onsets of blindness and cognitive abilities such as visuospatial working memory and intelligence. The auditory spatial processing was based on the “Bat-ears” echo sounds, which were novel to the participants. The experimental task involved sound localization among 15 positions, which demanded intense attention and was less easy to perform, whereas the control task required detection of a different pitch, which required low attention and was easy to perform. The different difficulty levels between the sound localization and pitch discrimination tasks may be a confounding factor in the results, since the major analysis was based on contrast subtraction. Future studies may consider using a psychophysical staircase procedure to control for the level of attention and other task taking processes (Collignon et al. 2011, 2013). The sound localization task appeared to be more difficult for the late- than early-onset blind individuals. Similarly, this would confound the between-group comparisons as difficult task would have called for more intense attention among the participants. Despite present findings did not confirm such possibility, future study should attempt to address this issue. Besides, our findings may not be comparable to those obtained from simple sound localization tasks. Last, but not least, the participants were not well trained on the sound localization task before the scanning. The participants may have employed other varied methods in response to the instructions given. This could increase the variances of the results and hence decrease the power of the group contrasts. Interpretation of the results should therefore be made with caution.

## Conclusion

This study explored how prior visual experience would modulate auditory spatial processing. Participants in the early- and late-blind groups were differed in terms of duration of living with an intact vision. Between-group analyses indicated dissociations of the right MOG, right SPG, and left SFG, which are likely attributable to the prior visual experience gained by the late-onset blind individuals. The right MOG played a significant role in auditory spatial processing among the early-onset blind individuals. In contrast, the left SFG contributed significantly to auditory spatial processing among the late-onset blind individuals. Prior visual experience modulates auditory spatial processing by means of enhancing the development of the visuospatial working memory for analyzing the spatial information embedded in the “Bat-ears” sounds and relating them to the different locations of the sound sources. Future studies should further manipulate the load on the visuospatial working memory and validate the role of SFG among the late-onset blind individuals.
